# An arthrogrypotic medical doctor with cervical kyphosis and thoracic lordoscoliosis

**DOI:** 10.1016/j.bonr.2016.11.002

**Published:** 2016-11-14

**Authors:** Manouchehr Safdarian, Mahdi Safdarian

**Affiliations:** Iran University of Medical Science, Tehran, Iran

**Keywords:** Arthrogryposis, Cervical kyphosis, Thoracic lordoscoliosis

## Abstract

Management of spinal deformities in a patient with arthrogryposis can be challenging for spine surgeons. The literature about the accompaniment of scoliosis; the most common spine deformity reported in arthrogryposis, is still poor. Moreover, the development of cervical kyphosis and thoracic lordoscoliosis in a patient with arthrogryposis is much rare. This paper reports a 26-year-old medical doctor with arthrogryposis who had underwent thoracic lordoscoliosis surgery about ten years ago with T6-L1 internal rods and is now presented with cervical kyphosis and C3-C4 cord stenosis, which made him a candidate for cervical cord decompression surgery.

## Highlights

• Arthrogryposis is a non-progressive congenital musculoskeletal disease.
• Arthrogryposis may be associated with different spinal deformities.
• The intelligence of arthrogrypotic patients should be considered to be normal to above normal.

## 1 Introduction

Arthrogryposis multiplex congenita is a group of congenital nonprogressive musculoskeletal disorders presenting with joints contracture in the vertebra and also in upper or lower limbs, leading to different degrees of flexion or extension limitations (Markus et al., 2012, Taricco and Aoki, 2009, Bamshad et al., 2009). Children born with arthrogryposis have abnormal muscle tissue fibrosis causing muscle shortening and disability in doing passive flexion or extension in the affected joints (Kalampokas et al., 2012). The frequency of the disorder is estimated to be about one person in every 3000 live births (Hall, 1985).

Scoliosis seems to be the most common spinal deformity associated with arthrogryposis. Here we report arthrogryposis in a 26-year-old medical doctor who have been developed cervical kyphosis and thoracic lordoscoliosis of spine in addition to his other musculoskeletal deformities.

## 2 Clinical presentation

**Fig. 1:**
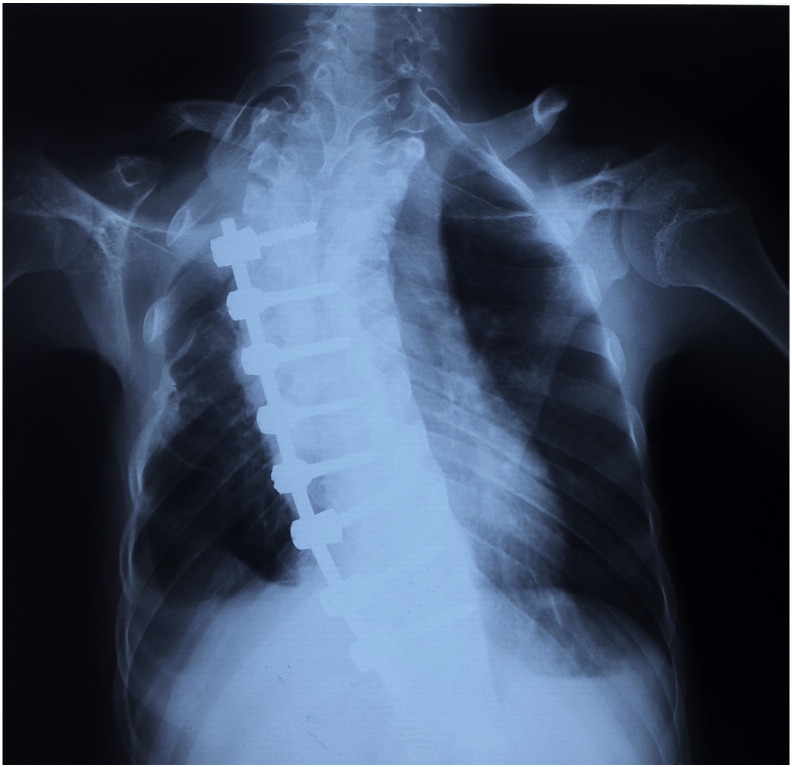
Thoracic lordoscoliosis reformed with T6-L1 internal rods.

This case report is written by the first author who is a 26-year-old medical doctor suffering from arthrogryposis (probably amyoplasia type) and was graduated from medical school last year. He was born with this congenital musculoskeletal disease. Some of his musculoskeletal deformities include left toes fibular deviation, left foot internal rotation, loss of left patella, shortness of left foot (9 cm in comparison to the right foot), flexion contracture of both knees, femoral heads and bilateral hips deformity, bilateral elbows flexion contracture, both wrists deformity, disability in left thumb extension, short stature, spinal lordoscoliosis and cervical kyphosis (with C3-C4 cord compression). This problems made him undergo many reformative surgeries during his life including bilateral Achilles tendons Z-plasty and legs casting for reforming deformities (1989), left hamstring muscle transfer (1998), left dislocated patella removal and left knee reformative surgery (2001), left femoral growth plate closure (2005), lordoscoliosis surgery with internal rods in T6-L1 (2005) (Fig. 1). Recently, he presented with cervical kyphosis and C3-C4 cord stenosis that made him a candidate for cervical spinal cord decompression and fixation.

He has no mental or psychological problem. He does not smoke, drink alcohol and take no specific medication. He has normal facies with no craniofacial abnormality. There is no one with similar problems in his relatives. His parents are cousins and he has five siblings who are completely healthy. Although his mother reports of a suspicious history of trauma in her pregnancy period.

**Fig. 2:**
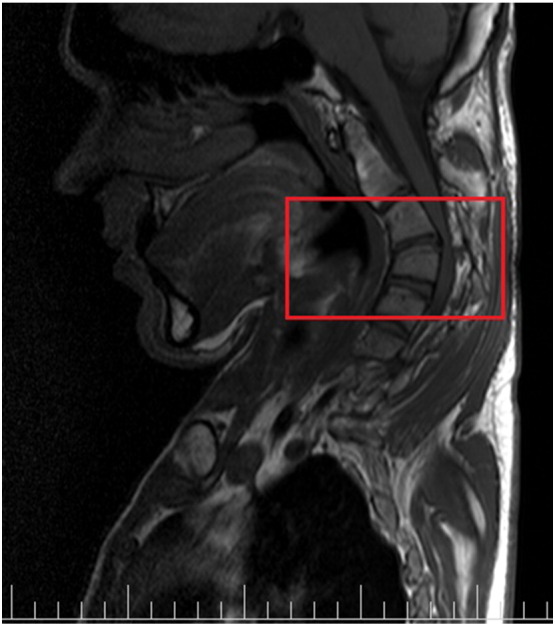
C3-C4 spinal cord compression in cervical MRI.

Last year, while studying hard for the residency examination after the graduation, he felt lots of pain in his neck in addition to paresthesia in hands and foots. He had a complaint of paresthesia for about two years but simultaneously with studying, it became very severe and made him stop studying. He consulted a neurosurgeon who found some neurological symptoms like hyperreflexia in addition to Babinski and Hoffman reflexes in the physical examination. He was referred for a total spine MRI, in which C3-C4 cord compression was reported (Fig. 2). After that, many neuro and orthopedic spine surgeons visited him, most of all suggested a cervical cord decompression and fixation surgery.

## 3 Discussion

Arthrogryposis is a not a diagnosis but a clinical finding. There are about twenty subtypes of arthrogryposis multiplex congenita based on clinical and genetic differences. Research has shown that there are more than thirty-five specific genetic sequence disorders associated with arthrogryposis (Bevan et al., 1978). The causes of arthrogryposis are not clarified yet but any factor that inhibits fetal normal joint movement can result in joint contractures (Bamshad et al., 2009).

There are three major groups of arthrogryposis; amyoplasia, distal arthrogryposis, and syndromic arthrogryposis. Amyoplasia is characterized by severe joint contractures and muscle weakness. Patients have a symmetrical joint or limb involvement with normal sensations. The intelligence is normal to above normal in children with amyoplasia (Bevan et al., 1978) however it is unknown how many of these children have an above normal intelligent. Distal arthrogryposis mainly involves the hands and feet and syndromic group includes a primary neurological or muscle disease (Bamshad et al., 2009).

The most common reported spinal deformity in arthrogryposis is scoliosis. Drummond and Mackenzie (1978) reported fourteen scoliosis in their 50 patients with arthrogryposis; an incidence of 28% (7) or Herron et al. reported eighteen scoliosis cases among eighty-eight arthrogryposis multiplex congenita which is about 20% (Herron et al., 1978). The accompaniment of arthrogryposis with other spinal deformities is reported scarcely in the literature. To our knowledge, this is the first time to report development of cervical kyphosis and thoracic lordoscoliosis in an arthrogrypotic patient. In addition, an arthrogrypotic patient receiving his medical diploma suffering from too many physical disabilities is the main instructive point of this case showing normal to above intelligence of these patients.

## 4 Conclusions

Arthrogryposis as a non-progressive congenital musculoskeletal disease may be associated with different spinal deformities which is reported less common in the literature. The intelligence of arthrogrypotic patients should be considered to be normal to above normal.

## Informed patient consent

The patient has consented to the submission of the case report for submission to the journal.
